# Clinical Profile and Outcome of Dengue Fever in Multidisciplinary Intensive Care Unit of a Tertiary Level Hospital in India

**DOI:** 10.5005/jp-journals-10071-23178

**Published:** 2019-06

**Authors:** Mahesha Padyana, Sunil Karanth, Shriram Vaidya, Justin Aryabhat Gopaldas

**Affiliations:** 1-4 Department of Critical Care Medicine, Manipal Hospital, Bengaluru, Karnataka, India

**Keywords:** APACHE, Dengue fever, SOFA, Severe dengue, Transaminitis

## Abstract

**Background:**

India is one of the seven identified countries in South-East Asia region regularly reporting dengue fever (DF)/dengue hemorrhagic fever (DHF) outbreaks. Even though the dengue prodrome and evolution of illness are most often similar in many patients, progress and outcome may differ significantly depending on the severity of illness as well as treatment instituted. We studied the clinical manifestations, outcome and factors predicting mortality of serology confirmed dengue fever cases admitted in Multidisciplinary Intensive Care Unit (MICU) of a high acuity healthcare facility in India.

**Methodology:**

All patients with serology proven dengue fever admitted to MICU between 1st July 2015 and1st December 2015 were included in the study. Clinical presentation, laboratory findings, severity of illness scores and outcome were recorded.

**Results:**

Majority of the patients (58.4%) belonged to 21–40 year age group. Hepatic (96.8%) followed by hematological (79.2%) involvement were the most common findings. CNS involvement observed among 27%. Survival to hospital discharge was 78.9%. Respiratory and gastrointestinal system involvement was associated with increased mortality. Acute respiratory distress syndrome (ARDS), acute kidney injury (AKI) and shock were the clinical syndromes associated with mortality. Serum lactate, aspartate transaminase (AST) and alanine transaminase (ALT) were significantly elevated among non survivors. Significant difference in sequential organ failure assessment (SOFA) and acute physiology and chronic health evaluation (APACHE) scores was also observed among survivors and non survivors.

**Conclusion:**

Organ system involvement and higher disease severity scores are strong predictors of mortality. High index of suspicion for atypical manifestations of dengue is warranted.

**How to cite this article:**

Padyana M, Karanth S, Vaidya S, Gopaldas JA. Clinical Profile and Outcome of Dengue Fever in Multidisciplinary Intensive Care Unit of a Tertiary Level Hospital in India. Indian J Crit Care Med 2019;23(6):270–273.

## INTRODUCTION

Dengue is a vector born tropical viral fever spread by *Aedes egypti* and *Aedes albopticus* mosquitoes. Incidence of dengue is rising in India from 6.34 per million population between 1998 and 2009 to 34.81 per million population between 2010 and 2014.^1^ Presentation of dengue fever can vary from malaise, fatigability as a part viral prodrome to shock and multiorgan dysfunction syndrome as a part of severe illness.^2^ Outcomes of patients with dengue fever admitted to critical care units have been studied less often. Prognostic factors which determine the clinical outcome of critically ill patients with dengue fever remain unclear.^3^

## METHODOLOGY

Retrospective single center study which included patients admitted to MICU with serology proven^4^ dengue fever with warning signs and severe dengue between 1st July and 1st December 2015 NS1-Antigen Strip® ELISA (BioRad), IgM and IgG- Capture ELISA Panbio® (Alere^TM^) were used for dengue serology testing. Patient management was as per WHO, Surviving Sepsis Campaign guidelines and established standards in intensive care practice. Demographics including age and gender distribution, signs and symptoms, SOFA and APACHE II scores were recorded. Outcome details including organ involvement and survival to hospital discharge were recorded. Statistical Analysis done using SPSS version 13.0 used for data analysis. Data analyzed by frequency, percentage, mean (±SD)/ Median (Inter Quartile Range- IQR). Logistic regression analysis for determination of factors predicting mortality.

## RESULTS

Total admissions to MICU during study period was 1,170 out of which patients with dengue fever were 96. Median length of ICU stay among severe dengue was 10 (IQR 14-8), dengue with warning sign was 4 (IQR 5-3) and dengue with no warning signs was 3 (IQR 5.75-2). Out of 96 patients with dengue, 47 (48.9%) were males and 49 were (51.1%) females. Patients belonged to age group between 21 years and 40 years were 58.4% (Table 1). Fifty-one patients had associated conditions like diabetes mellitus (18.5%), hypertension (14.4%), pregnancy (9.3%). Predominant symptom was fever (94.8%) followed by warning signs like vomiting (58.3%), breathlessness (57.3%), abdominal pain (41.7%), bleeding manifestations (31.3%) and altered sensorium (27.1%) (Table 2).

**Table 1:** Demographics

		*Number*	*%*
Age (in years)	<20	8	8.3%
	21 - 40	56	58.4%
	41 - 60	14	14.5%
	>60	18	18.8%
Sex	Males	47	49.0%
	Females	49	51.0%

**Table 2:** Clinical features

*Symptoms and signs*	*Frequency*	*%*
Fever	91	94.8%
Headache	39	40.6%
Bodyache	50	52.1%
Generalised weakness	75	78.9%
Loss of appetite	62	64.6%
Vomiting	56	58.3%
Abdominal pain	40	41.7%
Loose stools	20	20.8%
Breathlessness	55	57.3%
Bleeding manifestation	30	31.3%
Petichiae/echymosis	38	39.6%
Jaundice	16	16.7%
Altered sensorium	26	27.1%

**Fig. 1:**
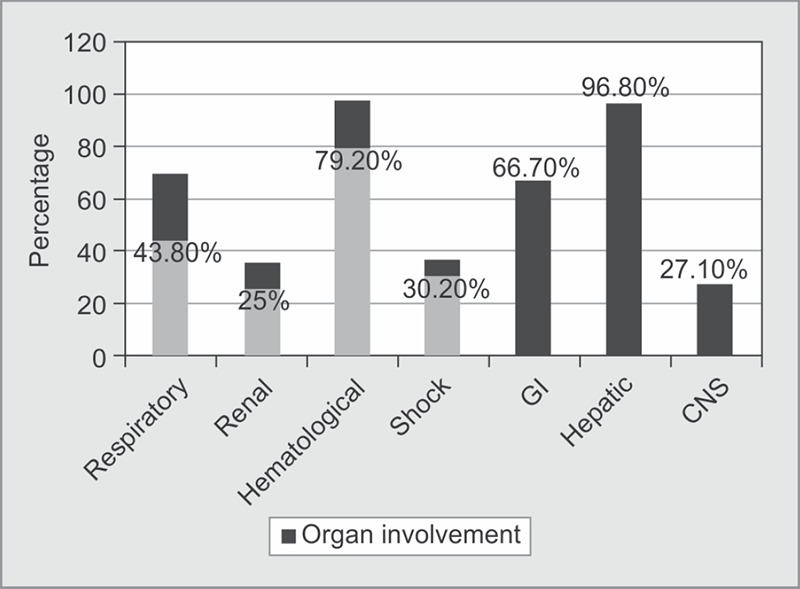
Organ involvement in patients during dengue fever

Predominant organ involvement was hepatic (transaminitis, jaundice) followed by gastrointestinal (abdominal pain, distension, loose stools, vomiting, ascitis) systems. Forty-four percent patients had gallbladder wall edema on ultrasound evaluation. Hematological involvement was predominantly thrombocytopenia and raised hematocrit. Respiratory system involvement had ARDS and pleural effusion. CNS manifestations included intracranial bleed, cerebral infarction and cerebellitis (in one patient). Patients with refractory shock requiring vasopressor initiation were 30.2% (Fig. 1). An average of 2 units of single donor platelet transfusion requirement per patient was noted in our study (Table 3).

Overall mortality was 21.1%. Among non survivors 12 (60%) females of which 8 belonged to 21–40 year age group. Median APACHE II score at 24 hours among survivors was 6.0 and non-survivors 17.5 (p <0.01). SOFA score at 48 hours among survivors and non survivors was 6 and 20, respectively (*p*<0.01) (Tables 4 to 6).

Total of 42 (43.8%) patients needed mechanical ventilation. Twenty-two of 76 patients (28.9%) who survived, required mechanical ventilation. Acute kidney injury was noted among 34 (35.4%) patients of which 24 (25%) needed either CRRT or SLEDD. Of all, 18.4% of survivors required renal replacement therapy and 29 patients (30.2%) required vasopressor support during ICU stay.

Secondary sepsis was noted in 16.5% of patients. Half of these were blood stream infections. Predominant organisms responsible are gram-negative (*Klebsiella pneumoniae* and *Acenetobacter baumanii*). Ten (13.1%) patients required tracheostomy for liberation of mechanical ventilation due to ICU acquired weakness (5.2%) and persistent neurological abnormalities like hemiparesis, ataxia, dysarthria, etc.

**Table 3:** Transfusion pattern

*Blood product*	*Mean no of units/patient*
Single donor platelet	2
Packed red blood cell	0.7
Fresh frozen plasma	1.2

**Table 4:** Mortality

	*Frequency*	*Percentage (%)*
Survivors	76	78.9
Non survivors	20	21.1

## DISCUSSION

Our study looked into epidemiological factors, organ involvement and mortality during ICU stay.

In our study, we noted that 50 patients (52.1%) had dengue with warning signs and 41 patients (42.7%) had severe dengue and only 5 patients (5.2%) had dengue without warning signs based on WHO classification.^4^ We observed that the mean age of our cohort of patients was 37 years. ICU requirement was significantly high among age group of 21-40 (58.4%). Similarly, in a study by Juneja et al. 39.6 year was the mean age of patients requiring ICU admission with dengue fever suggesting that younger age group is at higher risk compared to older.^5^ Vomiting, abdominal pain and bleeding manifestations were common warning signs which warranted ICU transfer, similar to observations in previous studies.^3,6^

Multiorgan involvement in dengue is well known. In our study we observed Hepatic involvement among 96.8%. AST and ALT were significantly high among non survivors. Similarly in a study by Nayak et al liver involvement was noted among 97.33%, AST and ALT were significantly high among dengue hemorrhagic fever and dengue shock syndrome group.^7^

Respiratory and gastrointestinal involvements had increased risk of mortality. AKI and moderate to severe ARDS (with PF ratio <200) was significantly high among non survivors. Similarly Shock and Lactates were also noted to be significantly high among non survivors. Study by Thanachartwet et al showed significantly high lactates among patients with organ failure and shock.^8^ In a study by Chen et al moderate ARDS (PF Ratio <200) followed by shock (SBP <90 mm Hg or MAP <65) and multiorgan failure was significant among non survivors.^3^ A study by Jain et al. had described liver and renal involvement, ARDS, Shock requiring vasopressor, metabolic acidosis as predictors of mortality.^9^ A study done in Cuttack, India had showed predominant organ failure was coagulation (48%). Respiratory failure was 19% and cardiovascular failure was 8%.^10^

**Table 5:** Factors associated with mortality

*Variable*	*Survivors (n = 76)*	*Non survivors (n = 20)*	*OR (IQR)*	*p value*
Jaundice	8 (10.5%)	8 (40%)	5.66 (1.78-18.0)	0.003
ARDS(PaO_2_/FiO_2_ <200)	22 (28.9%)	17 (85%)	13.9 (3.7-52.3)	<0.01
Acute kidney injury (AKI)	15 (19.7%)	19 (95%)	77.26 (9.56-624)	<0.01
Shock	16 (21%)	19 (95%)	71.3 (8.86-574)	<0.01
Central nervous system	20 (26.3%)	8 (40%)	1.86 (0.66-5.23)	0.235
Respiratory	48 (63.2%)	19 (95%)	11.08 (1.4-87.3)	<0.05
Gastrointestinal	45 (59.2%)	19 (95%)	13.1 (1.67-103)	<0.05

**Table 6:** Evaluation of variables in survivors and non survivors

*Variable*	*Survivors (n = 76)*	*Non survivors (n = 20)*	*p value*
Lactate (IQR)	3.0 (20-4.8)	13.4 (9.45-7.5)	<0.01
Aspertate Transaminase (IQR)	299.5 (148-1098.5)	4936 (2431.5-14340.3)	<0.01
Alanine Transaminase (IQR)	163 (83.75-469.5)	1412 (613-3832.75)	<0.01
Base Excess	-3.75 (-1.42-6.57)	-21.9 (-18.2- -24.0)	<0.01
Platelets (mean ±SD)	22493.4 (24405.3)	19200 (15949.3)	0.864
Total count (mean ±SD)	8258 (6032)	(13453 (10799.9)	0.095
Hematocrit (mean ±SD)	41.5 (9.84)	43.2 (9.54)	0.332
Serum albumin (g/dL), (mean ±SD)	2.67 (0.62)	2.44 (0.7)	0.264
APACHE median (IQR)	6.0 (4.0-9.0)	17.5 (9.5-35.75)	<0.01
SOFA 48 hours median (IQR)	6 (3.0-20.0)	20 (18-22)	<0.01

Neurological involvement may occur without associated bleeding manifestations, rash or thrombocytopenia. Neurological complications are reported to be rare in dengue as it is a non-neurotropic virus.^11^ However we observed that many patients (27.1%) were presented with altered sensorium as initial manifestation either due to primary CNS involvement (encephalopathy, encephalitis or intracranial bleed) or secondary to profound shock. Karoli et al described neurological involvement upto 14% in patients with dengue fever in the form of encephalopathy, hypokalemic periodic paralysis, myositis and Gullain-Barre syndrome. Another study by Nimmagadda et al placed incidence of neurological manifestations at 2% encephalitis or meningitis.^12,13^ In our study, three patients had infarct, two sustained intracranial hemorrhage, one had Posterior Reversible Encephalopathy Syndrome (PRES) and other was found to have cerebral abscess with hydrocephalus (overall incidence 8.3%). One of the patients with multiple infarcts had cerebrospinal fluid polymerase chain reaction (PCR) positive for dengue. Recently published data from Taiwan demonstrated that altered sensorium is an independent risk factor for mortality.^14^ However in our study, we did not find any significant association between altered sensorium and mortality.

Moratlity has been shown to be high among age group of >60 years in prior studies.^3^ Our study had younger patients between 21 years and 40 years requiring ICU and mortality was noted to be high among females between the age group of 21–40 years. In a retrospective cohort study from Brazil mortality of severe dengue was noted to be 3.1% over a period of 14 years. Age >55 year was associated with increased mortality.^15^ In a Chinese study, female sex and age group 30–39 years was associated with increased risk of dengue hemorrhagic fever and mortality especially with dengue 2 serotype^6^ Pang et al noted that adult patients belonging to 50 to 59 year age group or patients with diabetes had 5 times increased risk for ICU admission and increased mortality compared to patients who are younger than 30 year and without diabetes.^16^ Another study done in Singapore between 2007 and 2008 during dengue sero-type 2 epidemic observed that female sex, age group 30–49 years and presence of diabetes or hypertension were associated with increased risk of dengue hemorrhagic fever.^17^ However in our study, diabeties was not associated with significant increase in mortality.

There are certain limitations in our study. Interpretation of this study should be done bearing in mind that it is a retrospective single center study involving patients admitted in the intensive care unit. However, this study sheds light onto the factors which could predict mortality among patients admitted with dengue to critical care unit.

## CONCLUSION

Lactatemia, transaminitis, higher disease severity scores (APACHE II and SOFA), Shock, AKI and PaO_2_/FiO_2_ ratio less than 200 were associated with increased mortality. We advise high index of suspicion for atypical CNS manifestations in patients admitted with dengue fever to intensive care unit.
